# Dissecting the Origin of Breast Cancer Subtype Stem Cell and the Potential Mechanism of Malignant Transformation

**DOI:** 10.1371/journal.pone.0165001

**Published:** 2016-10-21

**Authors:** Xinyi Liu, Dongfei Feng, Dianming Liu, Shuyuan Wang, Xuexin Yu, Enyu Dai, Jing Wang, Lihong Wang, Wei Jiang

**Affiliations:** 1 College of Bioinformatics Science and Technology, Harbin Medical University, Harbin, China; 2 Department of Stomatology, Harbin Children's Hospital, Harbin, China; 3 Institute of Cancer Prevention and Treatment, Harbin Medical University, Harbin, China; Tianjin University, CHINA

## Abstract

**Background:**

Breast cancer is the most common incident form of cancer in women including different subtypes. Cancer stem cells (CSCs) have been confirmed to exist in breast cancer. But the research on the origin of breast cancer subtype stem cells (BCSSCs) is still inadequate.

**Methods:**

We identified the putative origin cells of BCSSCs through comparing gene signatures between BCSSCs and normal mammary cells from multiple perspectives: common signature, expression consistency, functional similarity and shortest path length. First, the potential origin cells were ranked according to these measures separately. Then Q statistic was employed to combine all rank lists into a unique list for each subtype, to prioritize the origin cells for each BCSSC. Next, we identified origin-related gene modules through integrating functional interaction network with differentially expressed genes. Finally, transcription factors of significant gene modules were predicted by Match^TM^.

**Results:**

The results showed that Luminal A CSC was most relevant to luminal progenitor cell or mature luminal cell; luminal B and HER2 CSC were most relevant to bipotent-enriched progenitor cell; basal-like CSC was most relevant to bipotent-enriched progenitor cell or mature luminal cell. Network modules analysis revealed genes related to mitochondrial respiratory chain (MRC) were significantly dysregulated during the origin of luminal B CSC. In addition, SOX10 emerged as a key regulator of MRC.

**Conclusions:**

Our study supports substantive evidence for the possible origin of four kinds of BCSSCs. Dysfunction of MRC may contribute to the origin of luminal B CSC. These findings may have important implications to treat and prevent breast cancer.

## Introduction

Cancer stem cells (CSCs) are a small subpopulation of cells inside tumors. They possess the capacity to self-renew and to cause the heterogeneous lineages of cancer cells that comprise tumors [1]. In the latest studies, the existence of CSCs has been validated in various kinds of cancer, such as colorectal cancer, bladder cancer and breast cancer [2–4]. Moreover they are responsible for tumorigenesis, recurrence, and metastasis [5]. Currently, cancer chemotherapy is cytotoxic to the bulk of tumor cells but fails to eliminate CSCs, thereby making them the leading reason for recurrence [6]. So, eradication of CSCs is the prerequisite of cancer therapy [7].

The origin of the CSC remains elusive. There are three hypotheses [8]: 1) CSCs derived from normal adult stem cells. Adult stem cells are long-lived cells with a high proliferative capacity tending to accumulate the mutations that lead to carcinogenesis [9]. 2) CSCs derived from progenitor cells. According to this hypothesis, through the mutation occurred in progenitor cells during the process of differentiation, they can obtain the ability of self-renewal, and then form cancer stem cells [10]. 3) CSCs derived from differentiated cells. For example, a combination of epidermal growth factor receptor (EGFR) pathway activation and loss of INK4A tumor-suppressor function induces a high-grade glioblastoma multiforme phenotype from differentiated astrocytes [11]. Different types of CSC may derive from different origin and lead to the formation of heterogeneous tumor. Lottaz *et al*. identified two putative founder cell populations for two subtypes of glioblastoma CSCs: fetal neural stem cell and adult neural stem cell [12].

Breast cancer is the most common incident form of cancer in women worldwide and responsible for 1.4 million new cases annually [13]. Perou *et al* classified breast cancers into 5 subtypes: luminal A, luminal B, HER2/Neu, basal-like and normal-like [14]. Each subtype has heterogeneous pathologies and clinical outcomes, suggesting the possibility that different subtypes of breast cancers may be derived from distinct breast cancer subtype stem cells (BCSSCs). However, it remains unclear whether different BCSSCs derived from different cells of origin. It will count for much to design personalized therapy if this key issue is clearly elucidated.

In this study, we compared the gene signatures of BCSSCs and normal mammary cells to identify the possible cellular origin of BCSSCs. Next we focused on the origin mechanism of luminal B CSC to further analyzed expression data of luminal B CSC and bipotent-enriched progenitor cell through a network module-based method in protein function interaction (FI) network. The results showed that genes related to the function of mitochondrial respiratory chain (MRC) were significantly dysregulated between normal and cancer condition. We also found SOX10 was the key regulator of genes associated with respiratory chain by transcription factor binding site analysis. Based on our data, we proposed a possible case of breast cancer subtype tumorigenesis that might explain the heterogeneity of breast cancer. These findings might have important implications for strategies to treat and prevent breast cancer.

## Methods

**Fig 1:**
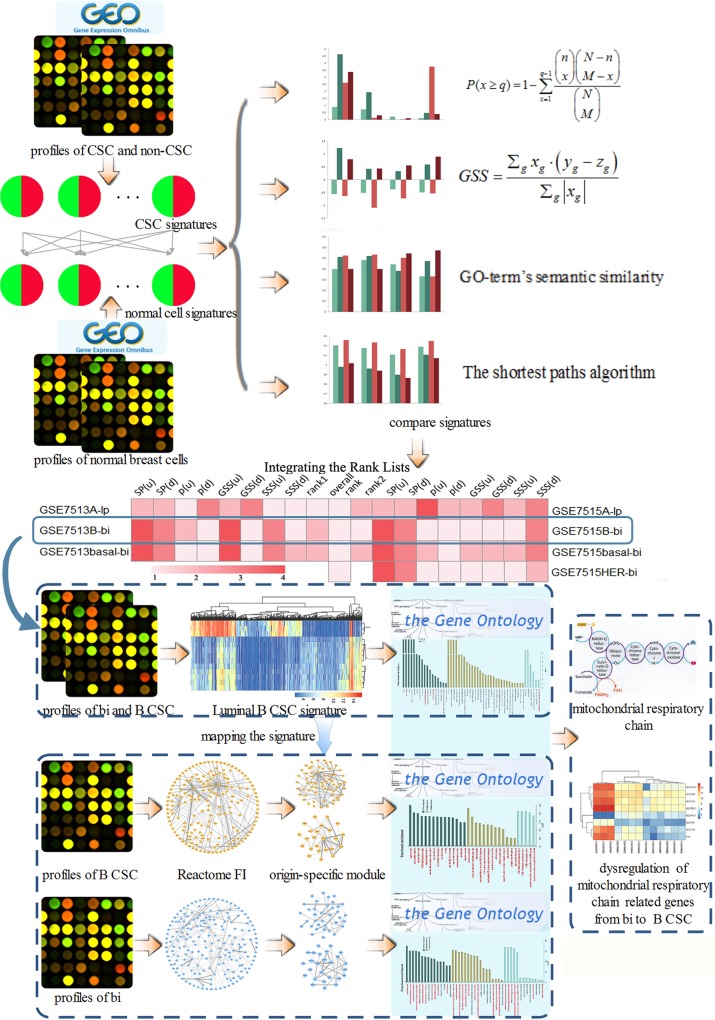
The work flow of our approach. Firstly, we downloaded gene expression profiles of normal mammary cells and BCSSCs data from GEO. We indentified signature genes for each type of normal mammary cells and BCSSCs. Secondly, we compared the gene signatures including enrichment analysis of signature gene sets, BCSSC GSS, GO-term’s SSS and the shortest path in HPRD network for up-regulated/over-expressed and down-regulated/under-expressed genes. We obtained eight rank lists of potential origin cells of BCSSCs for each expression profile. In order to prioritize the origin cells for each BCSSC, *Q* statistic was employed to combine all rank lists into a unique list. We discovered that luminal B subtype was most associated with bipotent-enriched progenitor cell. Next, we analyzed expression data of luminal B CSC and bipotent-enriched progenitor cell through a network module-based method in protein FI network. The result showed that genes related to the function of MRC were significantly dysregulated between normal and cancer condition. Bi, lp, ml and mb are abbreviation for bipotent-enriched progenitor cell, luminal progenitor cell, mature luminal and mature basal cell respectively. A, B, basal and HER are abbreviation for luminal A, luminal B, basal-like and HER2 subtypes, respectively.

We presented a computational pipeline to investigate the origin of BCSSCs through comparing the gene signatures of BCSSCs and normal mammary cells. We further analyzed the mechanism of malignant transformation from normal cells to BCSSCs through network module-based method in protein FI network. The workflow of our approach is shown in Fig 1.

### Preprocessing of gene expression profiles

The raw data (CEL files) of human breast cancer stem cells and normal mammary cell was downloaded from Gene Expression Omnibus (GEO) database. In this study breast cancer stem cells (GSE7513, GSE7515) [4] are classified into 4 subtypes using immunohistochemical results of estrogen receptor (ER), progesterone receptor (PR) and human epidermal growth factor receptor-2 (HER2)[15, 16]: luminal A subtype (ER/PR+, HER2-), luminal B subtype (ER/PR+, HER2+), basal-like subtype (ER-/PR-/HER2-) and HER2 subtype (ER-/PR-/HER2+). HER2 subtype was only compiled in GSE7515. All breast cancer stem cells include CSCs and non-CSCs expression profiles. Mature mammary epithelial cells are generated from progenitor cells and progenitor cells are generated from primitive bipotent cells through a hierarchical process. GSE11395[17] subdivide normal human mammary epithelial cells by surface markers including the expression profiles of bipotent-enriched progenitor cells (MUC1^-^CD133^-^ (CD10/THY1)^+^CD49f^+^) and committed luminal progenitor cells ((MUC1/CD133)^+^CD10^-^THY1^-^CD49f^+^) as well as mature luminal ((MUC1/CD133)^+^CD10^-^THY1^-^CD49f^-^) and myoepithelial cells (MUC1^-^CD133^-^ (CD10/THY1) CD49f^-^).

We normalized gene expression arrays using robust multiarray analysis (RMA) methods as implemented in the software BRB ArrayTools [18]. Based on log2-transformed data, two-sided *t* test was used to identify significant difference of gene expression between groups of samples. Meanwhile, we computed fold changes by comparing the mean gene expression levels between groups. In BCSSCs respect, we compared gene expression between each kind of BCSSC and corresponding non-CSC. We defined genes with significant *p* value (<0.05) and > 2 fold-change as up-regulated signature genes of each kind of BCSSC. On the other hand, genes with significant *p* value and < 0.5 fold-change were defined as down-regulated signature genes. In normal cells respect, we compared gene expression between each population and others. We defined genes with significant *p* value and > 2 fold-change as over-expressed signature genes of each kind of normal population. On the other hand, genes with significant *p* value and < 0.5 fold-change were defined as under-expressed signature genes.

### Enrichment analysis of signatures

The statistical significance of the overlaps between each kind of BCSSC and normal cell signature gene sets was calculated using the hypergeometric distribution. The *P* value of enrichment is calculated by

$$P(x\geq q)=1-\sum_{x=1}^{q-1}\frac{\left(\begin{array}{c} n\\ x \end{array}\right)\left(\begin{array}{c} N-n\\ M-x \end{array}\right)}{\left(\begin{array}{c} N\\ M \end{array}\right)},$$

where *n* is the total number of genes, *M* and *N* is the number of signature gene sets of BCSSCs and normal cells, respectively, and *x* is the number of genes in common. We conducted this analysis for each pair of normal over-expressed and BCSSC up-regulated signature genes as well as each pair of normal under-expressed and BCSSC down-regulated signature genes, respectively. Two cancer stem cell profiles were analyzed separately.

### Gene signature score (GSS)

For each normal mammary sample, a GSS was computed to measure the concordance of expression with each BCSSC. Higher score indicates the normal breast sample is more inclined to acquire the gene signature of the given BCSSC. GSS is defined as follow:

$$GSS=\frac{\sum_{g}x_{g}\cdot \left(y_{g}-z_{g}\right)}{\sum_{g}\left|x_{g}\right|},$$

where the sum is up-regulated or down-regulated genes in each BCSSC signature gene sets, *x*_*g*_ is the log2-fold-change of expression value for that gene in the BCSSC signature; *y*_*g*_ is log2-expression value for the same gene in one normal breast sample and *z*_*g*_ is log2-mean expression value of the same gene in all normal breast samples. We computed GSS for two cancer stem cell profiles separately.

### Semantic similarity score (SSS)

We evaluated the functional similarity between signatures of normal cell and BCSSC by gene ontology (GO) term’s semantic similarity. We calculated the SSS for each pair of normal over-expressed and BCSSC up-regulated signature genes as well as each pair of normal under-expressed and BCSSC down-regulated signature genes. We performed this analysis for two cancer stem cell profiles separately. This process was implemented by R package “GOSemSim” [19], which was developed to compute semantic similarity among GO terms, sets of GO terms, gene products and gene clusters based on the graph structure of GO. Here, we only used biological process category in GO, and selected “Lin” and “BMA” methods in “GOSemSim” to compute the similarity scores.

### Shortest path (SP) length

In protein-protein interaction network, Human Protein Reference Database (HPRD), we calculated the average length of the SP length between all possible pairs of nodes for normal over-expressed and BCSSC up-regulated signature genes or normal under-expressed and BCSSC down-regulated signature genes by R package “igraph”. We performed this analysis for two cancer stem cell profiles separately.

### Integration of the rank lists

We detected the relationship between signatures of normal cell and BCSSC from four different aspects including enrichment analysis, BCSSC gene signature score, GO term’s semantic similarity and shortest path for up-regulated/over-expressed and down-regulated/under-expressed signature genes. Consequently, we obtained eight rank lists of putative origins for each kind of BCSSCs according to these four values: *p*-value of hypergeometric distribution, GSS, SSS and the length of SP. In order to get an overall rank list for all pairs of normal cells and BCSSCs, we fused the eight separate rank lists using a *Q* statistic [20, 21]. The formula was as follows:

$$Q(r_{1},r_{2},\ldots ,r_{N})=N!V_{N}$$

$$V_{k}=\sum_{i=1}^{k}{\left(-1\right)}^{i-1}\frac{V_{k-i}}{i!}r_{N-k+1}^{i}$$

$$V_{0}=1,$$

where *N* representing number of separate rank lists used, and *r*_*i*_ is the rank ratio for the *i*-th rank list. Variable *V* is an intermediate variable.

### Identification of origin-specific gene modules

In order to further investigate the meaningful and biologically relevant subunits, we explored “origin-specific modules”. Here, we identified modules whose genes were both topologically close in network and highly correlated in expression level by a cytoscape plug-in, Reactome FI [22]. The method first calculated the Pearson correlation coefficients (PCCs) among all FI pairs in the gene expression data set, and then assigned the PCCs to the edges of the FI network. Next, it used a highly efficient network clustering algorithm to cluster the weighted network into a series of gene interaction modules. We applied this approach to gene expression data of luminal B CSC and bipotent-enriched progenitor cell, respectively.

For the modules obtained we firstly filtered the modules passed to the next step of analysis by removing whose number of nodes was smaller than default threshold, and which had an average PCC below 0.5. Second, for the filtered list of modules, we mapped “origin-specific signature” genes to find out significantly enriched modules (hypergeometric distribution test *p*<0.05). Next, we investigated overlapped genes between CSC and normal cells to find modules that shared common “origin-specific signature” genes.

### Prediction of transcription factor

In order to study the upstream regulatory elements, we conducted prediction of transcription factor by Match^TM^ that used the matrix library collected in TRANSFAC [23]. We obtained transcripts of genes from UCSC. 2000bp promoter upstream sequence of each transcript was extracted as the transcription factor binding regions. We applied minimize false positive rate (minFP) option and set core similarity cut-off to 1 to acquire the strictest output.

## Results

### Identification of gene signatures in normal mammary cells or BCSSCs

To gain insight into the molecular characteristics of normal and malignant breast cells, we downloaded gene expression profiles of normal mammary cells and BCSSCs data from GEO. In normal mammary cells comparison (one population vs. others), 138, 218, 66 and 333 genes were significantly under-expressed in bipotent progenitor cells, committed luminal progenitor cells, mature luminal and myoepithelial cells, respectively. 307, 283, 314 and 318 genes were significantly over-expressed in bipotent progenitor cells, committed luminal progenitor cells, mature luminal and myoepithelial cells, respectively (*t*-test *p*<0.05 and two-fold differentially expressed, Table 1, details in methods section). We defined the significantly differentially expressed genes of each kind of normal cells as signature gene sets, including under- and over-expressed genes. In BCSSCs comparison, we compared gene expression in two BCSSCs expression profiles separately for each subtype (CSCs vs. non-CSCs) using the same criteria. In the respect of up-regulated genes, we identified 361, 270 and 286 genes in GSE7513 for luminal A, luminal B, basal-like CSCs respectively and 218, 379, 875 and 448 genes in GSE7515 for luminal A, luminal B, basal-like and HER2 CSCs respectively. In the respect of down-regulated genes, we identified 188, 890 and 129 genes in GSE7513 for luminal A, luminal B, basal-like CSCs respectively and 199, 1251, 931 and 1115 genes in GSE7515 for luminal A, luminal B, basal-like and HER2 CSCs respectively (Table 2, details in methods section). Up-regulated and down-regulated genes together were the signature gene sets of BCSSCs. So far we defined signature gene sets for each kind of normal mammary cell and BCSSC.

**Table 1:** Number of normal cells signature genes.

cell population	over-expressed	under-expressed
bipotent progenitor cells	307	138
luminal progenitor cells	283	218
mature luminal cells	314	66
myoepithelial cells	318	333

**Table 2:** Number of BCSSCs signature genes.

subtypes	up-regulated		down-regulated	
	GSE7515	GSE7513	GSE7515	GSE7513
luminal A	218	361	199	188
luminal B	379	270	1251	890
basal-like	875	286	931	129
HER2	448		1115	

### Detection of BCSSC origin through integrating the characteristic of signatures

We investigated the correlation between the signatures of each kind of normal mammary cell and the signatures of each kind of BCSSC in two expression profiles. First, hypergeometric distribution was employed to conduct enrichment analysis. We compared BCSSC up-regulated with normal cell over-expressed signature genes and BCSSC down-regulated with normal cell under-expressed signature genes separately (S1A Fig). Lower *p*-value represented that two gene sets shared more signature genes. Next, we computed the GSS of BCSSC for each normal cell population to further explore the correlation on gene expression level (details in methods section, S1B Fig). We also calculated the GSS using up-regulated and down-regulated BCSSC signature genes separately. The higher GSS indicated the up-regulated BCSSC signature genes tended to be more highly expressed in a given normal cell, or the down-regulated BCSSC signature genes were more lowly expressed in this normal cell.

Next, we evaluated the functional similarity of signatures genes. GO-term’s semantic similarity has been proposed for comparing genes at the functional level on the basis of GO annotation [24]. A higher value of SSS indicated a greater functional similarity between signatures of BCSSC and normal cells (details in methods section, S1C Fig).

Finally, we compared signatures on the network topological structure level. The shortest paths algorithm has been applied to discover novel candidate disease related genes through known disease related genes in the protein-protein interaction network [25, 26]. The SP between two nodes was the smallest distance value in their corresponding paths [27]. A smaller value of SP indicated a stronger relevance between signatures of BCSSC and normal cells (details in methods section, S1D Fig).

**Fig 2:**
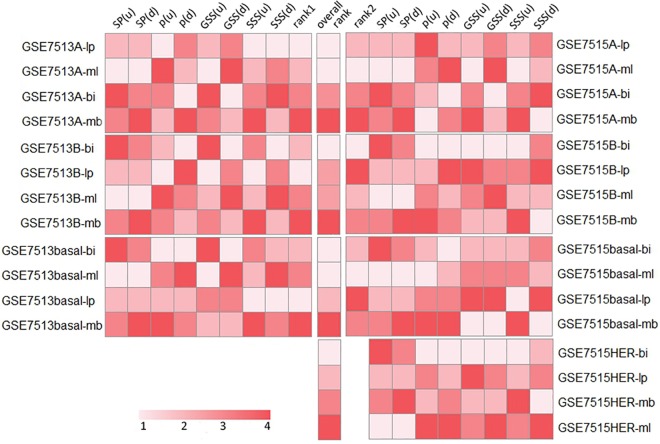
The rank lists for four subtypes of breast cancer. The rank lists were integrated by *Q* statistic. Rank 1 and rank 2 were the integrated rank list for GSE7513 and GSE7515, separately. Overall rank was the average of rank 1 and rank 2. U, up-regulated/over-expressed genes; d, down-regulated/under-expressed genes. Colored bar indicates the ranking of each pair of relationship. The lighter presents the higher ranking; the darker presents the lower ranking.

We ranked the eight values (*p*-values of hypergeometric distribution, GSS, SSS and SP for up-regulated/over-expressed and down-regulated/under-expressed signature genes) for each kind of breast cancer subtype and four normal mammary cells. We integrated the eight rank lists for each breast cancer expression profiles by *Q* statistic [21] and implemented by Endeavour program [20]. Two rank lists were obtained for each breast cancer subtype: one for GSE7513, another for GSE7515. HER2 subtype only had one rank order because it was only compiled in GSE7515. The average of these two rank orders was called the overall rank. Top of the final list was the possible cellular origin for each kind of BCSSC. The results showed that luminal A CSC was most relevant to luminal progenitor cell or mature luminal cell; luminal B and HER2 CSC were most relevant to bipotent-enriched progenitor cell; basal-like CSC was most relevant to bipotent-enriched progenitor cell or mature luminal cell (Fig 2). The luminal A subtype is ER-positive and the least aggressive breast cancer molecular subtype [28] and might arise through ER-positive progenitors such as luminal progenitor cells [29]. Some researchers indicated that luminal B tumors might derive from ER-negative progenitor cell, which could undergo luminal lineage differentiation and display a positive expression of ER [30]. In addition, luminal B, basal-like and HER2 subtypes all had the possibility to origin from bipotent-enriched progenitor cell as the result showed. It’s credible because there existed common genomic alterations in basal-like, HER2 and luminal B breast tumors suggesting a common cell origin of these tumor subtypes [29].

### Functional analysis of differentially expressed genes between luminal B CSC and bipotent-enriched progenitor cell

**Fig 3:**
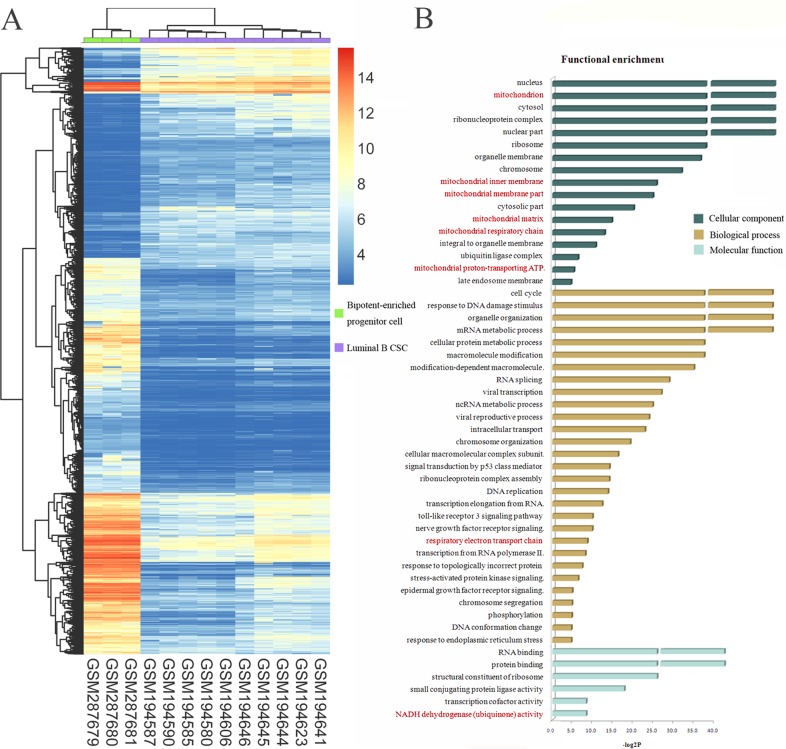
Differentially expressed genes between luminal B CSC and bipotent-enriched progenitor cell. **A**, heat map of differentially expressed genes between luminal B CSC and bipotent-enriched progenitor cell. Each row represents a gene; each column, a sample. Colored bar indicates the expression level. **B**, the result of functional enrichment analysis of differentially expressed genes based on GO. The terms in red are related to mitochondrion. The disconnected bar represents *p*-value of the term is zero.

Basal-like and HER2-enriched subtypes displayed the worst prognosis followed by luminal B, whereas luminal A has a good prognosis [31]. However, luminal B tumors often have the worst long-term prognosis, as exhibiting rapid recurrence after endocrine therapy compared to basal-like and HER2-enriched tumors [32]. Thus, we focused on this origin of luminal B subtype in the following analysis. In order to investigate the potential mechanism of malignant transformation, we detected the difference in gene expression patterns between luminal B CSC and bipotent-enriched progenitor cell (luminal B CSC vs. bipotent-enriched progenitor cell). 6033 overlapped differentially expressed genes (1999 up-regulated and 4034 down-regulated) between GSE7513 and GSE7515 were detected (the same criteria as above, Fig 3A) termed “origin-specific signature”.

We next conducted functional enrichment analysis of the entire “origin-specific signature” genes based on GO [33]. In order to reduce the redundancy to find more statistically and biologically meaningful function, we chose R package “GOFuntion” to implement the analysis [34]. The majority of significantly enriched biological process terms included cell cycle, response to DNA damage stimulus, signal transduction by p53 class mediator and DNA replication, which reflected known properties of stem cells and breast cancer (Fig 3B). It was interesting to note that substantial portions of cellular component terms were associated with mitochondrion. In addition, MRC was also one of significantly enriched terms of biological process and molecular function. This phenomenon arrested our attention. We discovered that numerous studies have suggested that mitochondrial processes might play important roles in tumor initiation and progression. Mitochondrial dysfunction clones of breast cancer cells exhibited higher migration and invasive behaviors compared with their parental cells [35]. Increased incidence of mtDNA polymorphisms were detected in breast cancer samples compared with normal samples [36]. These results indicated that MRC function may play a key role in promoting transformation from bipotent-enriched progenitor cell to luminal B CSC. We should focus on it in following study.

### Identification of origin-specific gene modules

In order to further investigate the meaningful and biologically relevant subunits, we explored “origin-specific modules”, that is, dysfunction of these modules might cause malignant transformation. Topological properties of protein FI network have been studied to extract new disease-related knowledge [37], and co-expressed genes of protein-protein interaction network are highly connected indicating that they are functionally related [38]. Here, we identified modules whose genes were both topologically close in network and highly correlated in expression level by a cytoscape plug-in, Reactome FI. We applied this approach to gene expression data of luminal B CSC and bipotent-enriched progenitor cell, respectively.

**Fig 4:**
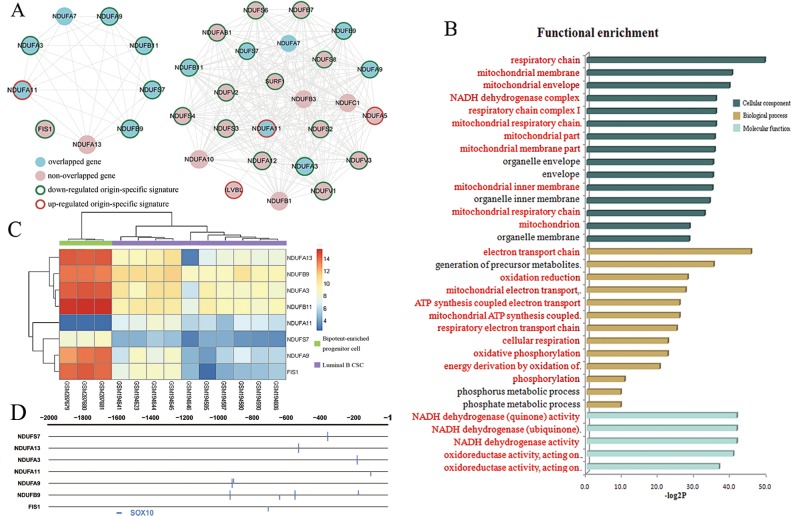
Identification of origin-specific gene modules. **A**, module 1 from luminal B CSC (left) and module 3 from bipotent-enriched progenitor cell (right). **B**, the result of functional enrichment analysis of module 1. The terms in red are related to mitochondrion. **C**, heat map of genes in module 1. Each row represents a gene; each column, a sample. Colored bar indicates the expression level. **D**, the result of prediction of transcription factor for module 1 by Match^TM^. Each row represents a transcript. The number indicates the upstream distance from transcription start site.

Two modules for each condition were obtained after filtration (details in methods section). Module 1 from luminal B CSC and module 3 from bipotent-enriched progenitor cell shared common “origin-specific signature” genes (Fig 4A, S1 and S2 Tables); module 2 from luminal B CSC and module 4 from bipotent-enriched progenitor cell shared common “origin-specific signature” genes (S2 Fig).

We performed functional enrichment analysis for four modules by DAVID. An amazing discovery for us was that module 1 and module 3 were significantly enriched in MRC function on all three GO categories (*p*<0.05, Fig 4B and S3 Fig). Basically all terms associated with MRC including electron transport chain, cellular respiration and ATP synthesis coupled electron transport were enriched. It is incredibly impressive that this result showed astonishing consistency with what we detected above.

The common “origin-specific signature” genes shared by module 1 and 3 were virtually all down-regulated in luminal B CSC except NDUFA11 (Fig 4C). It have been previously described that a low amount of MRC complexes was well adapted to the low energy demand of this G0-G1 resting stem cell type [39]. Li LD, *et al* further discovered that NDUFA9 played a pivotal role in stem cell self-renewal and cancer growth because it uniquely expressed in human embryonal carcinoma cells comparing with human embryonic stem cells [40]. Moreover, NDUFB9 was down-regulated in highly metastatic breast cancer cells compared with parental breast cancer cell line which indicating that NDUFB9 promoted breast cancer cells proliferation, migration and invasion [41]. On the other hand, inefficient MRC function has been implicated in the vicious cycle of reactive oxygen species (ROS) production which increased risk of breast cancer [42]. Moreover, human mammary carcinoma cells were shown to have depressed expression levels of complex I (NADH-ubiquinone oxidoreductase) genes, the transfer of electrons from NADH to respiratory chain [43]. These previous discoveries confirmed our results. The gene and complex involvement of MRC function in stem cell and breast cancer implied its pivotal role in the process of the origination of luminal B CSC. So we defined module 1 of cancer state as “origin-specific module”.

### Transcriptional regulation of origin-specific module

In order to study the upstream regulatory elements, we conducted prediction of transcription factor for module 1 by Match^TM^. We obtained nine transcripts for nine genes of module 1. As a result, we found the transcription factor binding site of SOX10 existed in the promoters of seven transcripts in module 1 (Fig 4D). SOX10 was validated as a sensitive diagnostic marker for breast cancer [44] as well as a marker and principal regulator of neural crest stem cells (NCSCs) playing an important role in the maintenance and migration of NCSCs [45]. In addition, it was also to be confirmed that SOX10 is peripherally associated with the mitochondrial outer membrane and raise the possibility of signal transduction cascade between the nucleus and mitochondria [46]. In conclusion, SOX10 is associated with breast cancer, stem cell and mitochondria function. Our findings corroborated these discoveries from another point of view suggesting dysregulation of SOX10 might facilitate dysfunction of MRC, thus prompting malignant transformation of bipotent-enriched progenitor cell.

## Discussion

The identification and comprehension of breast CSCs is rapidly progressing recent years. Some have postulated that eradication of CSCs is the prerequisite of cure for cancer because they are responsible for tumorigenesis, recurrence, and metastasis [5]. But the fundamental question that which kind of normal cell is the key cellular target for malignant transformation is still unknown. Here, this present work attempted to provide a broad view of the origin of CSC of distinct breast cancer subtypes through a high throughput manner.

We compare the signatures between BCSSCs and their putative normal cells of origin from different aspects including enrichment analysis, BCSSC gene signature score, GO-term’s semantic similarity and the shortest path in HPRD network for up-regulated/over-expressed and down-regulated/under-expressed signature genes. We finally obtained eight distinct rank lists for each BCSSC gene expression profile. Through integrating the rank lists, we found the relationships associated with bipotent-enriched progenitor cell ranked frequently at the top of the list. This result is reasonable considering the known characteristic of progenitor cell. Bipotent-enriched progenitor cell was long-lived cells possessing pluripotency. Mutations in tumor suppressor genes or oncogenes were incidental which might affect differentiation potential of progenitor cells that drove tumor phenotypes [47].

The relationship of luminal B CSC and bipotent-enriched progenitor cell ranked higher than other origin pairs. In fact, the overlapped genes between luminal B CSC and bipotent-enriched progenitor cell signature indeed had stem cell related function. For example, ST7 and USP11, two of the overlapped up-regulated/over-expressed genes between luminal B CSC and bipotent-enriched progenitor cell, existed in gene set that represented the core expression signature of embryonic stem (ES) cells. Function of this gene set reflected the activity of the regulatory pathways associated with their stem-like character [48]. Furthermore, five down-regulated/under-expressed overlapped genes (EPS8, IRX5, MYO5B, GUCY1A3 and TNFRSF1B) were in the gene set representing under-expressed genes bounding by Polycomb Repressive Complex 2 in human ES cells [49].

The “origin-specific module” we found through network modules analysis was significantly associated with MRC function. Mitochondrion is a critical organelle in eukaryocyte. It has been established that mitochondria burn the calories in our diet with the oxygen to make energy and heat to maintain our body temperature through ATP synthesis. All enzymes and coenzymes involved in this process constitute MRC. As a by-product of energy production, the mitochondria also generate the endogenous reactive oxygen species (ROS) which is crucial not only in redox signaling for many cellular events, but also to be pivotal in variety of harmful oxidative damage under pathological conditions [50, 51]. The low gene expression of MRC complexes is requisite for existence of stem cell type [39]. But in the meantime, it also produces more ROS production which increased risk of breast cancer [42]. It is accord with our result that genes of MRC are down-regulated in luminal B CSC. The phenomenon that lower expression value of these genes in luminal B CSC compared with progenitor cell maybe implies that CSCs are less differentiated cells, which means their growth pattern and tendency to spread is more reckless. Finally, transcription factors such as SOX10 which directly targeted mitochondria-associated genes may represent new targets for the elimination or modulation of luminal B CSC.

There are also some other studies of possible mechanisms about origin of luminal B CSC. Previous research revealed that histone acetyltransferase KAT6A played key role in maintaining the ability of self-renewal and activity of luminal breast cancer stem cell [52]. Bone morphogenetic protein (BMP) pathway and Wnt pathway was also reported to be the prominent mechanisms of quiescence and acquisition of stemness in luminal breast cancer cell lines [53]. The association between these possible mechanisms should be further discussed to ascertain the specific mechanism of origin of CSC.

## Conclusion

The data of our study support substantive evidence for the possible origin of four kinds of BCSSCs and the mechanism of malignant transition from bipotent-enriched progenitor cells to luminal B CSCs. These discoveries is striking, since, in spite of dysfunction of MRC has been confirmed during cancer development, the research disclosed its key role in origin of cancer stem cell is absent. But further expression profile studies will be necessary to further determine the origin of breast tumor phenotypes. Currently limited gene expression data of breast cancer stem cell was the biggest restrictions of our study. Our discoveries also need to be confirmed by low-throughput experiments. In the longer term, detailed characterization of the modules active or repressive in CSCs is likely to yield powerful diagnostic and prognostic markers and, quite possibly, the upstream regulatory elements will be the attractive targets for eliminating CSCs to prevent recurrence and improve survival in breast cancer patients.

## Supporting Information

S1 Fig Comparing gene signatures of normal mammary cells and BCSSCs. **A**, the result of hypergeometric distribution. **B**, BCSSC gene signature scores (GSS) for each cell population in each normal sample. **C**, the semantic similarity score (SSS) for each pair of BCSSC and normal cell signature. **D**, the shortest path lengths (SP) between signatures of normal cells and BCSSCs.
(TIF)

S2 Fig Module 2 from luminal B CSC (left) and module 4 from bipotent-enriched progenitor cell (right). (TIF)

S3 Fig The result of functional enrichment analysis of module 3. The functional enrichment analysis was conducted by GO. The terms in red are related to mitochondrion.
(TIF)

S1 Table Genes in module 1. (DOCX)

S2 Table Genes in module 3. (DOCX)
