# Supplementary figures and images for: Dissecting the Origin of Breast Cancer Subtype Stem Cell and the Potential Mechanism of Malignant Transformation
Source: PLoS One. 2016 Oct 21;11(10):e0165001. doi: 10.1371/journal.pone.0165001 (PMC5074511; doi:10.1371/journal.pone.0165001)

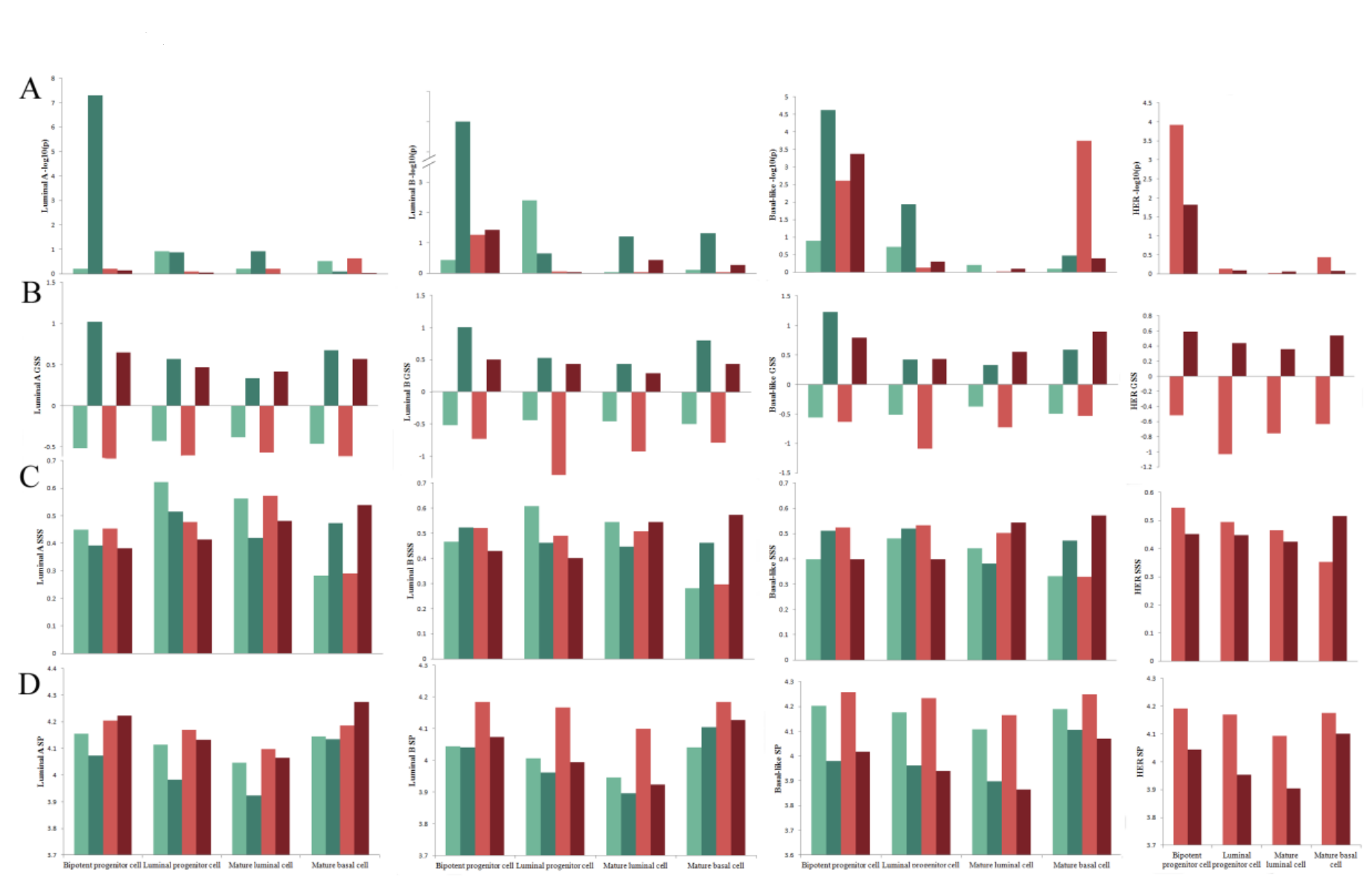

Supplement: S1 Fig — A, the result of hypergeometric distribution. B, BCSSC gene signature scores (GSS) for each cell population in each normal sample. C, the semantic similarity score (SSS) for each pair of BCSSC and normal cell signature. D, the shortest path lengths (SP) between signatures of normal cells and BCSSCs. (TIF) [file pone.0165001.s001.tif]

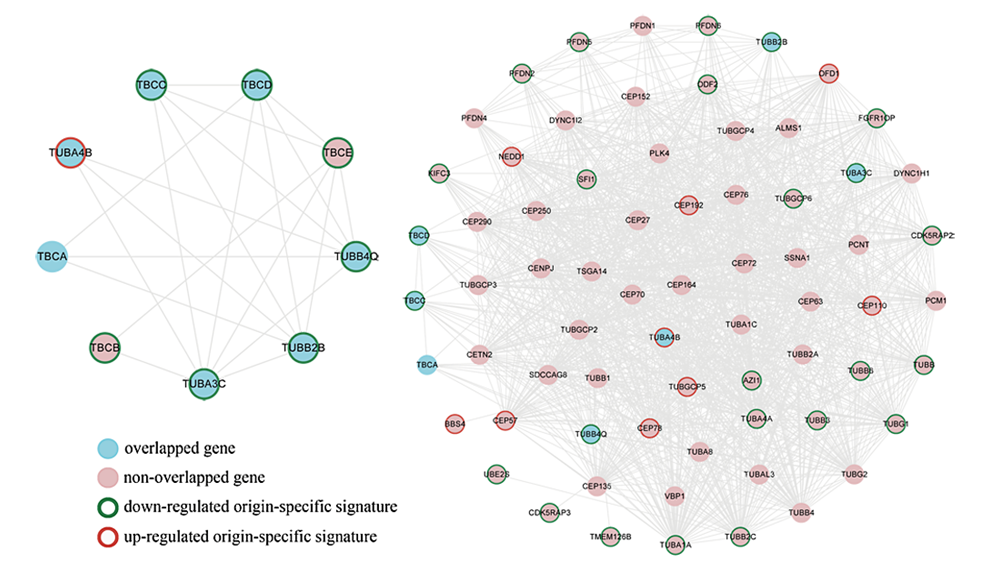

Supplement: S2 Fig — (TIF) [file pone.0165001.s002.tif]

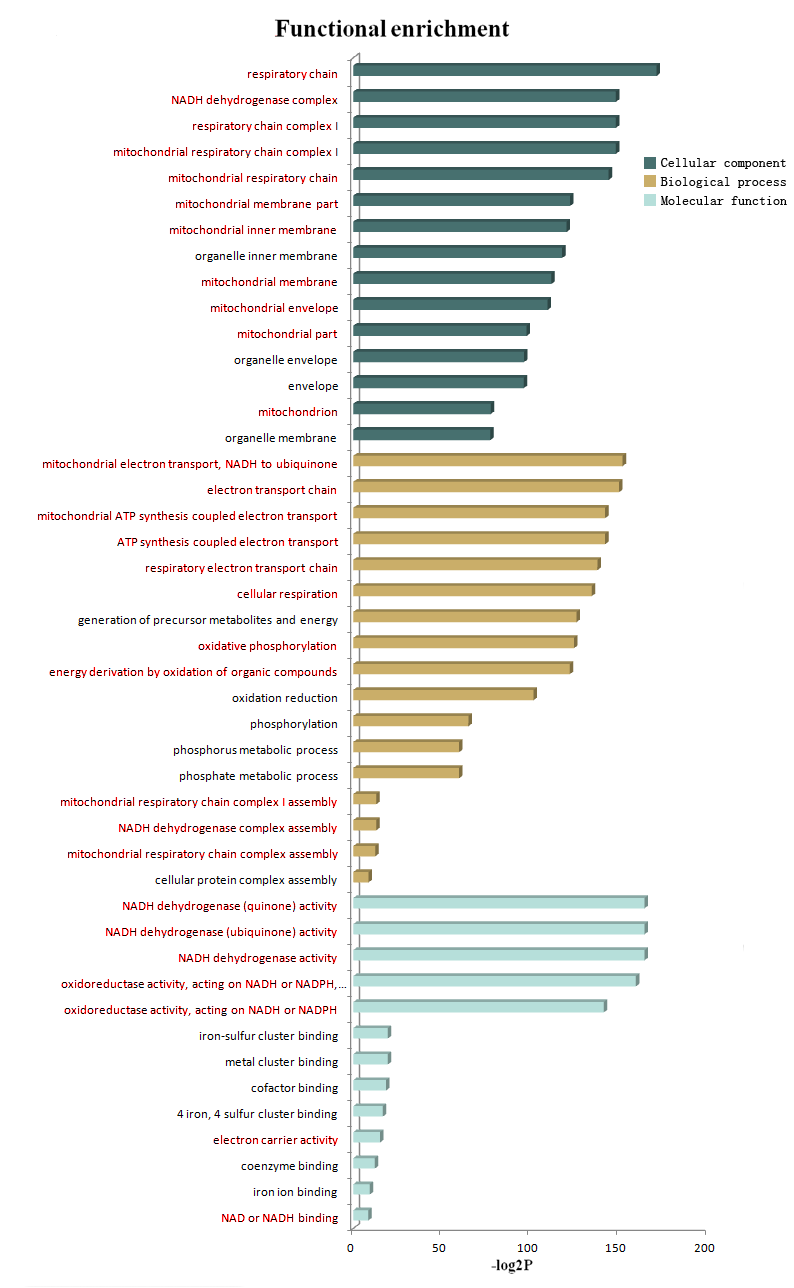

Supplement: S3 Fig — The functional enrichment analysis was conducted by GO. The terms in red are related to mitochondrion. (TIF) [file pone.0165001.s003.tif]
