# Supplementary material for: Dissecting the Origin of Breast Cancer Subtype Stem Cell and the Potential Mechanism of Malignant Transformation
Source: PLoS One. 2016 Oct 21;11(10):e0165001. doi: 10.1371/journal.pone.0165001 (PMC5074511; doi:10.1371/journal.pone.0165001)
Supplement: S1 Table — (DOCX) [file pone.0165001.s004.docx]

**S1 Table. Genes in module 1**

| Gene | Expression pattern |
| --- | --- |
| FIS1  NDUFA11*  NDUFA13  NDUFA3*  NDUFA7*  NDUFA9*  NDUFB11*  NDUFB9*  NDUFS7* | down-regulated  up-regulated  NULL  down-regulated  NULL  down-regulated  down-regulated  down-regulated  down-regulated |

*The overlapped genes between signatures of luminal B CSC and bipotent-enriched progenitor cells. Expression pattern represents the difference in gene expression between luminal B CSC and bipotent-enriched progenitor cells.
