# Supplementary material for: Dissecting the Origin of Breast Cancer Subtype Stem Cell and the Potential Mechanism of Malignant Transformation
Source: PLoS One. 2016 Oct 21;11(10):e0165001. doi: 10.1371/journal.pone.0165001 (PMC5074511; doi:10.1371/journal.pone.0165001)
Supplement: S2 Table — (DOCX) [file pone.0165001.s005.docx]

**S2 Table. Genes in module 3**

| Gene | Expression pattern |
| --- | --- |
| ILVBL  NDUFA10  NDUFA11*  NDUFA12  NDUFA3*  NDUFA5  NDUFA7*  NDUFA9*  NDUFAB1  NDUFB1  NDUFB11*  NDUFB3  NDUFB7  NDUFB9*  NDUFC1  NDUFS2  NDUFS3  NDUFS4  NDUFS6  NDUFS7*  NDUFS8  NDUFV1  NDUFV2  NDUFV3  SURF1 | up-regulated  NULL  up-regulated  down-regulated  down-regulated  up-regulated  NULL  down-regulated  down-regulated  NULL  down-regulated  NULL  down-regulated  down-regulated  NULL  down-regulated  down-regulated  down-regulated  down-regulated  down-regulated  down-regulated  down-regulated  down-regulated  down-regulated  down-regulated |

*The overlapped genes between signatures of luminal B CSC and bipotent-enriched progenitor cells. Expression pattern represents the difference in gene expression between luminal B CSC and bipotent-enriched progenitor cells.
